# Influence of body mass index on survival in indolent and mantle cell lymphomas: analysis of the StiL NHL1 trial

**DOI:** 10.1007/s00277-017-3003-0

**Published:** 2017-04-30

**Authors:** Lukas Weiss, Thomas Melchardt, Alexander Egle, Georg Hopfinger, Hubert Hackl, Richard Greil, Juergen Barth, Mathias Rummel

**Affiliations:** 1 Salzburg Cancer Research Institute, Department of Internal Medicine III, Salzburg Cancer Research Institute, Cancer Cluster Salzburg, Paracelsus Medical University, Muellner Hauptstrasse 48, 5020 Salzburg, Austria; 2 Department of Internal Medicine I, Medical University of Vienna, Vienna, Austria; 3 Division of Bioinformatics, Medical University of Innsbruck, Innsbruck, Austria; 4 Medizinische Klinik IV, Hospital of the Justus-Liebig-University, Giessen, Germany

**Keywords:** Body mass index, BMI, Lymphoma, NHL, Prognosis, B-symptoms

## Abstract

Obesity is a well-known risk factor for the development of cancer, but its influence on the course of disease is still controversial. We investigated the influence of body mass index (BMI) on overall survival (OS) in 502 patients with indolent non-Hodgkin’s lymphoma or mantle cell lymphoma in a subgroup analysis of the StiL (Study Group Indolent Lymphomas) NHL1 trial. We defined a cut-off of 22.55 kg/m^2^ by ROC calculation and Youden Index analysis and stratified patients into “low BMI” and “high BMI”. Five-year OS was significantly longer in the high BMI group (82.2%) when compared to that of the low BMI group (66.2%) (HR 0.597; 95%CI 0.370–0.963; *p* = 0.034). BMI was also an independent prognostic factor for OS in multivariate analysis (HR 0.541; 95%CI 0.332–0.883; *p* = 0.014). Of note, patients had a significantly lower BMI in the presence than patients in the absence of B-symptoms (*p* = 0.025). BMI significantly impacts on OS in indolent non-Hodgkin’s lymphoma and mantle cell lymphoma, which may be influenced by the effect of B-symptoms on BMI.

**Electronic supplementary material:**

The online version of this article (doi:10.1007/s00277-017-3003-0) contains supplementary material, which is available to authorized users.

## Introduction

Obesity is not only a well-known risk factor for the development of cardiovascular disease and diabetes [1], but also for the development of several types of cancer [2], including lymphomas [3]. Despite this known negative general impact of obesity on morbidity as well as mortality, we [4] and others [5] have described the somehow unexpected improved survival of overweight and obese patients with diffuse large B cell lymphoma, although others could not reproduce this finding [6].

Considering the present prevalence of excess body weight in the western hemisphere and its continuing global increase [7], medical oncologists are frequently confronted with overweight and obese patients, including their specific set of comorbidities [1] and differential pharmacokinetics [8]. More and more scientific effort is put into deciphering the effects of obesity once a cancer diagnosis has been established.

For hematological malignancies, several studies could not demonstrate a negative impact of overweight and obesity on survival: a retrospective analysis of 712 patients with B cell non-Hodgkin’s lymphoma (NHL) treated with chemotherapy showed no negative impact of higher BMI on OS or PFS [9].

Underdosing of chemotherapy is frequently seen in patients with a calculated body surface area (BSA) of more than 2 m^2^ [10]. Furthermore, a possible influence of B-symptoms on BMI has been unknown so far, but is highly probable, since unexplained weight loss of more than 10% of body weight within the last 6 months constitutes one of its defining criteria [11].

The StiL (Study Group Indolent Lymphomas) NHL 1 trial was a prospective, muticenter, randomized, controlled phase III trial to compare the efficacy and tolerability of chemo-immunotherapy with bendamustine and rituximab (BR) versus R-CHOP in patients with previously untreated indolent non-Hodgkin’s lymphoma or mantle cell lymphoma [12]. This trial was conducted by the StiL and could demonstrate a significantly longer median progression-free survival (PFS) for BR with 69.5 months compared to 31 months in the R-CHOP arm (*p* < 0.0001; HR 0.58). Since BR was also associated with significantly lower toxicity than R-CHOP, it has since then been widely adopted as new standard first-line regimen for patients with indolent NHL, as well as patients with mantle cell lymphoma who are not eligible for intensive therapy [13].

We performed an unplanned subgroup analysis of 502 patients of the StiL NHL1 trial, intended to investigate the influence of BMI on the OS in a well-defined study cohort of patients with indolent NHL and mantle cell lymphoma.

## Methods

As required by the StiL NHL1 protocol, patients aged 18 years or older with a WHO performance status of 2 or less were eligible for inclusion if they had a histologically confirmed diagnosis of mantle cell lymphoma or indolent non-Hodgkin’s lymphoma (follicular (grades 1 and 2), lymphoplasmacytic (Waldenstrom’s macroglobulinemia), small lymphocytic, and marginal-zone lymphoma). All patients had to have a previously untreated stage III or IV disease with indication for therapy. For detailed inclusion and exclusion criteria, see Rummel et al. [12]. All procedures followed were in accordance with the ethical standards of the responsible committee on human experimentation (institutional and national) and with the Helsinki Declaration. Of the 549 patients initially included in the study, 35 were excluded due to protocol violations and for another 12 patients, no biometric data was available, leading to 502 patients eligible for this unplanned subgroup analysis. The BMI was calculated as weight (kg) divided by the square of height (m). The patients were stratified into BMI groups according to the WHO guidelines: underweight (BMI <18.5 kg/m^2^), normal weight (BMI 18.5 to <25 kg/m^2^), overweight (BMI 25 to <30 kg/m^2^), obesity class I (BMI 30 to <35 kg/m^2^), obesity class II (BMI 35 to <40 kg/m^2^), and obesity class III (≥40 kg/m^2^) [14]. The optimal BMI cut-off for discerning the life and death status of patients at the end of the follow-up time was calculated based on the receiver operating characteristics (ROC) analyses and the Youden Index J, which represents the maximum of sensitivity_c_ + specitivity_c_-1 for all cut points in the ROC curve [15]. The robustness of the BMI cut-off was tested by 1000 bootstrap iterations, and a 95% confidence interval was provided. The height and weight were consistently recorded at screening. Mann-Whitney *U* test and Pearson’s *χ*2 test were used for univariate analyses of baseline characteristics, where appropriate. An association of the different parameters with BMI groups was also tested with a multivariate logistic regression analysis. The survival was estimated using Kaplan-Meier curve analysis, with statistical comparison using the log-rank statistic. A two-tailed significance level of 0.05 was considered statistically significant. Only statistically significant factors were included into multivariate Cox regression analysis. The BMI was also considered as continuous variable and the association with OS or PFS was tested with a Cox regression analysis. In either case, the Cox proportional hazard assumption was tested and time-varying effects on the hazard were analyzed using Schoenfeld residuals. All statistical analyses were carried out using the IBM® SPSS® statistics software (version 20) and the statistal environment R (including packages survival, OptimalCutpoints, boot, and ROCR).

## Results

### Patient characteristics

We performed an unplanned subgroup analysis of 502 patients with indolent non-Hodgkin’s lymphoma (NHL) or mantle cell lymphoma included in the StiL NHL1 trial. All patients were previously untreated, and height and weight were consistently recorded at study entry. The mean BMI for the whole cohort was 26.3 ± 4.2 kg/m^2^ SD with a range from 15.4 to 43.6 kg/m^2^. The majority of patients were of normal weight (40.8%) or overweight (40.2%), whereas only 1.2% were underweight and 17.7% obese (Supplemental Figure 1). These values are comparable to the general German population with a similar distribution within the BMI categories, but considerably differ from the reported mean BMI of 28.4 kg/m^2^ for the general population in the USA [7].

This analysis was not dominated by extreme outliers since only 6 patients were underweight (<18.5 kg/m^2^), 17 patients were classified into obesity class II (BMI 35 to <40 kg/m^2^), and only 3 patients into obesity class III (≥40 kg/m^2^).

The age, type of treatment, percentage of mantle cell histology, International Prognostic Index (IPI) score, and mean lactate dehydrogenase (LDH) levels were well balanced between the two BMI groups, but there was a trend for the presence of B-symptoms (*p* = 0.063) and poorer performance status (*p* = 0.074) in the low BMI group (Table 1). Of note, there were significantly more female patients (*p* = 0.002) and significantly more patients with stage IV disease (*p* = 0.006) in the low BMI group. Multivariate logistic analysis of baseline parameters confirmed sex (*p* = 0.0048) and stage (*p* = 0.0003) as the only parameters showing a significant difference between the BMI groups.

**Table 1:** Patient characteristics stratified by BMI

	Overall	Low BMI (<22.55 kg/m2)	High BMI (>22.55 kg/m2)	*P* value
	*N = *502	*N = *89	*N = *413	
BMI				
Mean ± SD	26.32 ± 4.23	20.93 ± 1.39	27.48 ± 3.70	
Range	15.39–43.55	15.39–22.53	22.57–43.55	
Age (years)				
Mean ± SD	62.1 ± 10.3	60.7 ± 11.0	62.4 ± 10.2	0.163^a^
Range	31–83	35–79	31–83	
> 60 years (%)	61.4	56.2	62.5	
Sex (%)				
Male	53.0	38.2	56.2	0.002^b^
Female	47.0	61.8	43.8	
Treatment (%)				
BR	50.8	46.1	51.8	0.326^b^
R-CHOP	49.2	53.9	48.2	
Histology (%)				
Mantle cell lymphoma	18.3	14.6	19.1	0.318^b^
Follicular lymphoma	54.2	47.2	55.7	0.145^b^
Other histologies	27.5	38.2	25.2	0.013^b^
Stage (%)				
II	3.0	4.5	2.7	0.006^b^
III	19.0	3.4	22.3	
IV	77.6	92.1	74.5	
Missing (*N*/total)	1/502	0/89	1/413	
Performance status (%)				
0	39.7	33.7	41.0	0.074^b^
1	54.3	56.2	53.9	
2	6.0	10.1	5.1	
Missing (*N*/total)	3/502	0/89	3/413	
IPI (%)				
Low (0–1)	20.4	22.4	20.0	0.809^b^
Low–intermediate (2)	42.2	38.8	43.0	
High–intermediate (3)	28.2	27.1	28.4	
High (4–5)	9.2	11.8	8.6	
Missing (*N*/total)	12/502	4/89	8/413	
B-Symptoms (%)				
Yes	34.2	42.7	32.2	0.063^b^
Missing (*N*/total)	2/502	0/89	2/413	
LDH (U/mL)				
Mean ± SD	243 ± 110	244 ± 106	243 ± 110	0.927^a^

^a^Mann-Whitney *U* test

^b^Pearson’s chi-square test

### Higher BMI is associated with significantly longer overall survival

We could observe a certain dose effect of BMI on OS: at 5 years, 76.3% of patients with normal weight were alive, compared to 78.9% of overweight and 87.6% of obese patients. This dose effect could also be found in the two largest patient subgroups of follicular lymphoma and mantle cell lymphoma. In order to evaluate the influence of BMI on OS and for better comparability with other parameters, we defined a cut-off of 22.55 kg/m^2^ by ROC analyses and Youden Index J (see “Methods”). A 95% confidence interval of the cut-off was calculated based on 1000 bootstrap iterations (21.97 to 28.57 kg/m^2^).

**Fig. 1:**
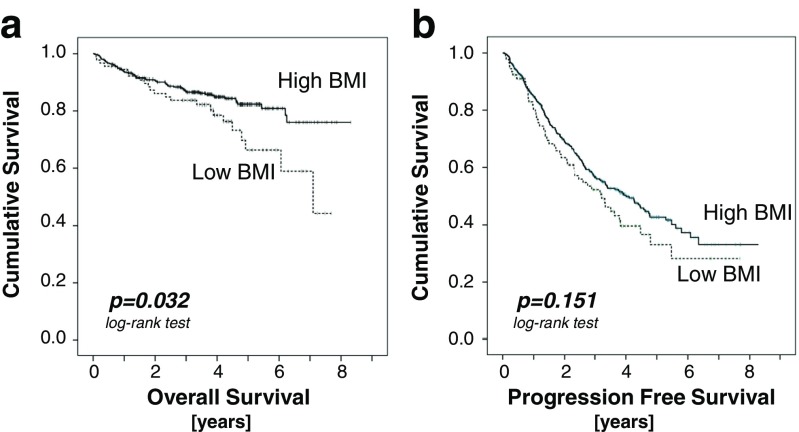
**a** Overall survival split by BMI: Patients with higher BMI (>22.55 kg/m^2^; *n* = 413) had significantly longer OS than patients with lower BMI (<22.55 kg/m^2^; *n* = 89). **b** Progression-free survival split by BMI: No significant difference was observed between the two groups

The patients with higher BMI had significantly longer OS (HR 0.597; 95%CI 0.370–0.963; *p* = 0.034) with 82.2% of patients alive at 5 years versus 66.2% in the low BMI group (Fig. 1a). The median OS was not reached at 8.3 years in the high BMI group but was 7.3 years in the low BMI group. During the observation period, 62 out of 413 patients died in the high BMI group, whereas 23 out of 89 patients died in the low BMI group. The median follow-up for OS was 4.0 years. After excluding patients with mantle cell lymphoma, we analyzed OS in the remaining 409 patients with indolent NHL: the patients in the high BMI group showed longer OS with 85.0% of patients alive at 5 years versus 70.2% in the low BMI group (HR 0.581; 95%CI 0.331–1.022; *p* = 0.059).

Of interest, there was a slight trend for longer PFS in the high BMI group when compared to the low BMI group, which however did not reach statistical significance (HR 0.798; 95%CI 0.586–1.087; *p* = 0.152), with 42.5% of patients without progression after 5 years in the high BMI group versus 32.9% in the low BMI group (Fig. 1b). During the observation period, 190 out of 409 patients progressed in the high BMI group, whereas 51 out of 89 patients progressed in the low BMI group. The median follow-up for PFS was 4.0 years.

BMI was also considered as a continuous variable and we could not observe a significant association with OS (*p* = 0.24) or PFS (*p* = 0.71) using Cox regression analysis. Proportional hazard assumptions were tested based on Schoenfeld residuals and were not violated for OS (*p* = 0.13) or PFS (*p* = 0.91). The effect of BMI on the hazard is only sligthly changing over the log of OS times. Furthermore, it is only sligthly changing up to 3 years of PFS times and then linearly increasing, thus might be fitted by a quadratic function.

### BMI is an independent predictor of overall survival

To evaluate the importance of BMI compared to established prognostic markers, we performed a Cox regression analysis. BMI as well as age, World Health Organization (WHO) performance status, IPI score, B-symptoms, LDH levels, and mantle cell lymphoma histology had significant prognostic power for OS in univariate analysis (Table 2), whereas sex, stage, and type of treatment did not. All significant parameters were further included into multivariate analysis. The age, BMI, WHO performance status, and mantle cell lymphoma histology remained significant and independent prognostic factors in multivariate testing, whereas IPI score, B-symptoms, and LDH levels lost their significance. Furthermore, we could not observe a significant prognostic role of statistical interaction between BMI and treatment for OS (*p* = 0.063) using Cox regression analysis with interaction term.

**Table 2:** Cox regression analysis

	Univariate				Multivariate			
	*N*	HR	95% CI	*p* value	*N*	HR	95% CI	*p* value
Age								
≤60 yrs vs. >60 yrs	502	3.248	1.859–5.675	<0.001	487	2.509	1.326–4.746	0.005
Sex								
male vs. female	502	0.913	0.595–1.400	0.676			not included	
BMI								
<22.55 vs. >22.55	502	0.597	0.370–0.963	0.034	487	0.541	0.332–0.883	0.014
Stage								
II or III vs. IV	501	1.618	0.896–2.921	0.111			not included	
Performance status								
0–1 vs. 2	499	4.188	2.392–7.331	<0.001	487	2.831	1.539–5.208	0.001
IPI score								
0–2 vs. 3–5	490	2.948	1.905–4.563	<0.001	487	1.168	0.622–2.193	0.628
B-symptoms								
Yes vs. no	500	1.721	1.121–2.643	0.013	487	1.185	0.747–1.879	0.472
LDH level								
≤240 U/mL vs. >240 U/mL	494	2.198	1.435–3.366	<0.001	487	1.682	0.971–2.915	0.064
Histology								
Mantle cell vs. other	502	2.155	1.356–3.424	0.001	487	1.662	1.014–2.726	0.044
Treatment								
BR vs. R-CHOP	502	1.040	0.680–1.592	0.855			not included	

### Dose capping in overweight and obese patients

**Fig. 2:**
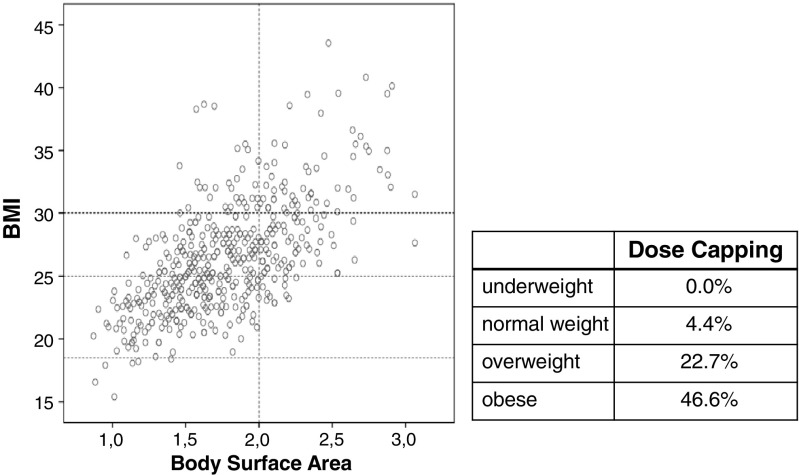
Dose capping and BMI: A substantial portion of overweight and obese patients have experienced dose capping to a body surface area of 2 m^2^. Dot plot depicting the correlation between BMI and body surface area, each *dot* representing a single patient. The accompanying *table* indicates the percentages of patients experiencing dose capping in the respective BMI subgroup

Dosing of chemotherapy is based on the patient’s body surface area whose calculation is based on individual height and weight. Similarly, BMI is calculated by individual height and weight and therefore is positively correlated with BSA. In the StiL NHL1 study cohort, no underweight patient and only 4.4% of normal weight patients experienced dose capping to a BSA of 2 m^2^, whereas as many as 22.7% of overweight and 46.6% of obese patients experienced a priori dose reduction in this fashion (Fig. 2). These high numbers could be observed although no deviating dosing algorithms for overweight or obese patients have been designated by the study protocol. In overweight and obese patients, (*N* = 291) we could not evidence a significant difference in OS with or without dose capping (HR 0.639; 95%CI 0.316–1.291; *p* = 0.212). Furthermore, in the overweight/obese subgroup, dose capping led to significantly lower rates of grade 3/4 leukopenia (41.4%) when compared to patients without dose capping (55.4%; *p* = 0.004). The rates of anemia and thrombopenia were generally low (<2.5%) and differences therefore not deemed clinically relevant. In patients with a BSA over 2 m^2^, we could not observe a significant difference in the rate of dose capping in the BR arm (65.2%) when compared to the R-CHOP arm (73.6%; *p* = 0.284).

### B-symptoms and BMI

**Fig. 3:**
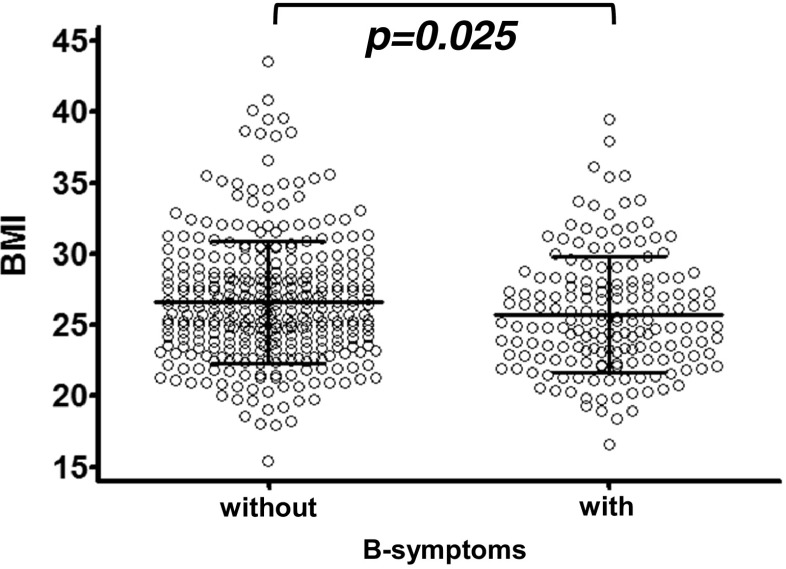
BMI and B-symptoms: Patients had a significantly lower BMI in the presence than in the absence of B-symptoms. Each *dot* corresponds to a single patient, *error bars* representing the mean and standard deviation

B-symptoms are a symptom complex consisting of fever of more than 38 °C, night sweats, and unexplained weight loss of more than 10% of body weight within the last 6 months [11]. As a consequence, B-symptoms may also impact on body weight and thereby BMI. The presence of B-symptoms has been recorded at study entry, but retrospectively cannot be attributed to one of the three defining criteria. In our analysis, patients with B-symptoms showed a significantly shorter median PFS with 3.0 years compared to 4.4 years in the absence of B-symptoms (log-rank *p* = 0.018). Furthermore, patients with B-symptoms had significantly shorter OS (log-rank *p* = 0.012) with 83.8% of patients alive at 5 years versus 71.1% in the absence of B-symptoms. Interestingly, the patients had a significantly lower BMI in the presence (mean 25.7 kg/m^2^) than in the absence of B-symptoms (mean 26.6 kg/m^2^) (*p* = 0.025, Fig. 3). This holds true for all patients with indolent NHL (*N* = 409; *p* = 0.019) as well as the subgroup of follicular lymphoma (*N* = 271; *p* = 0.022). However, we were not able to detect a significant difference in patients with mantle cell lymphoma with (*N* = 35; mean 26.0 kg/m^2^) or without (*N* = 56; mean 26.4 kg/m^2^) B-symptoms (*p* = 0.729). So, although the existing data cannot dissect whether B-symptomatic patients have experienced substantial weight loss and do not provide information on longitudinal weight changes, it nevertheless shows a significant difference in BMI.

## Discussion

Analyzing 502 patients treated in the StiL NHL1 trial, we could detect a significant association of higher BMI (>22.55 kg/m^2^) with longer OS. Although this finding is in line with reports in diffuse large B cell lymphoma, where overweight and obese patients showed longer OS [4, 5], it is somehow unexpected: obesity in general is associated with higher morbidity and mortality [1] and is known to promote a state of low-level chronic inflammation [16]. Adipokines play a role in inflammation [17] and are often increased in obese patients. As an example, leptin increases proliferation in hematopoietic cells [18], circulating monocytes [19] as well as T lymphocytes [20]. Polymorphisms in the genes encoding leptin and leptin receptors are associated with an increased risk of NHL [21]. Furthermore, insulin and the insulin-like growth factor 1—both being increased in the plasma of obese patients [2]—have been shown to induce cell proliferation and to inhibit apoptosis [22]. An important role of the IGF-1/IGF-1R for proliferation and survival of malignant cells was described in mulitple myeloma [23], mantle cell lymphoma [24] as well as Hodgkin’s lymphoma [25].

So, although there is mounting evidence that obesity is associated with an increased risk for the development of lymphoma [3], surprisingly, obesity does not seem to negatively impact on the further course of disease.

Several other factors may contribute to this observed BMI effect, one of them being B-symptoms: In most clinical trials for indolent NHL, around one third of patients are reported to have B-symptoms [12, 26]. Although B-symptoms per se represent an indication for treatment, it is very likely that many patients with yet undiagnosed lymphoma or those with established diagnosis but unrecognized or milder, “subclinical” B-symptoms, experience considerable weight loss until the initiation of first lymphoma-specific therapy. Here, a relevant difference in the biology of lymphomas has to be noted, with follicular lymphoma showing a mean time to first treatment of 3 years [27, 28], in contrast to aggressive lymphomas such as diffuse large B cell lymphoma, where the first diagnosis itself is equivalent to indication for treatment. Therefore we hypothesize that in indolent NHL, B-symptoms can significantly impact on BMI, which is supported by our observation of significantly lower BMI in B-symptomatic patients with indolent NHL, but not in patients with mantle cell lymphoma—a subtype known to generally exhibit a more aggressive clinical course than indolent NHL.

But also pharmakokinetics have to be taken into account when analyzing the effect of BMI: in aggressive lymphomas, higher body weight has been associated with an increased rituximab clearance leading to shorter rituximab exposure times when compared to lower body weight [29].

Dosing of chemotherapy is based on the patient’s BSA, and higher BSA frequently coincides with higher BMI. Despite existing literature confirming the safety and clinical necessity of full weight-based chemotherapy dosing, patients with excess body weight frequently receive limited chemotherapy doses in daily practice [10]. A substantial portion of overweight and obese patients in the StiL NHL1 trial have also experienced dose capping to a BSA of 2 m^2^, although being treated in a randomized phase III trial. This practice clearly contradicts current treatment guidelines [30] and is explicitly addressed in the follow-up protocol of the NHL7 protocol of the StiL group.

In our opinion, the present study has an important strength: we evaluated a considerable cohort of 502 patients treated within a prospective, randomized phase III clinical trial, thereby ensuring uniform data collection of biometric, histological, and clinical parameters.

In conclusion, our study shows that excess body weight may affect the clinical course of lymphomas. To our minds, the most pressing open question remains to what extent B-symptoms might bias the interpretation of the individual contribution of BMI. Despite the breath-taking evolution of our understanding of the molecular basis of lymphomas, thanks to technologies such as next-generation sequencing, we should not neglect the clinical methodologies such as taking an accurate patient’s history or standardized assessment and documentation of biometric data. Only the collection of molecular as well as clinical data according to the highest standards of scientific quality will allow us to link a genotype to a certain phenotype and thereby possibly generate new insights into disease biology.

## Electronic supplementary material

Figure 1 BMI distribution: Of 502 patients 1.2% were underweight with a BMI of <18.5 kg/m2 (under), 40.8% were of normal weight with a BMI of ≥18.5 – 24.9 kg/m2 (normal), 40.4% were overweight with a BMI of ≥25 – 29.9 kg/m2 (over) and 17.5% were obese with a BMI ≥30 kg/m2 (obese). (PPT 72 kb)
