# Supplementary figures and images for: Influence of body mass index on survival in indolent and mantle cell lymphomas: analysis of the StiL NHL1 trial
Source: Ann Hematol. 2017 Apr 30;96(7):1155–62. doi: 10.1007/s00277-017-3003-0 (PMC5486799; doi:10.1007/s00277-017-3003-0)

## Slide 1
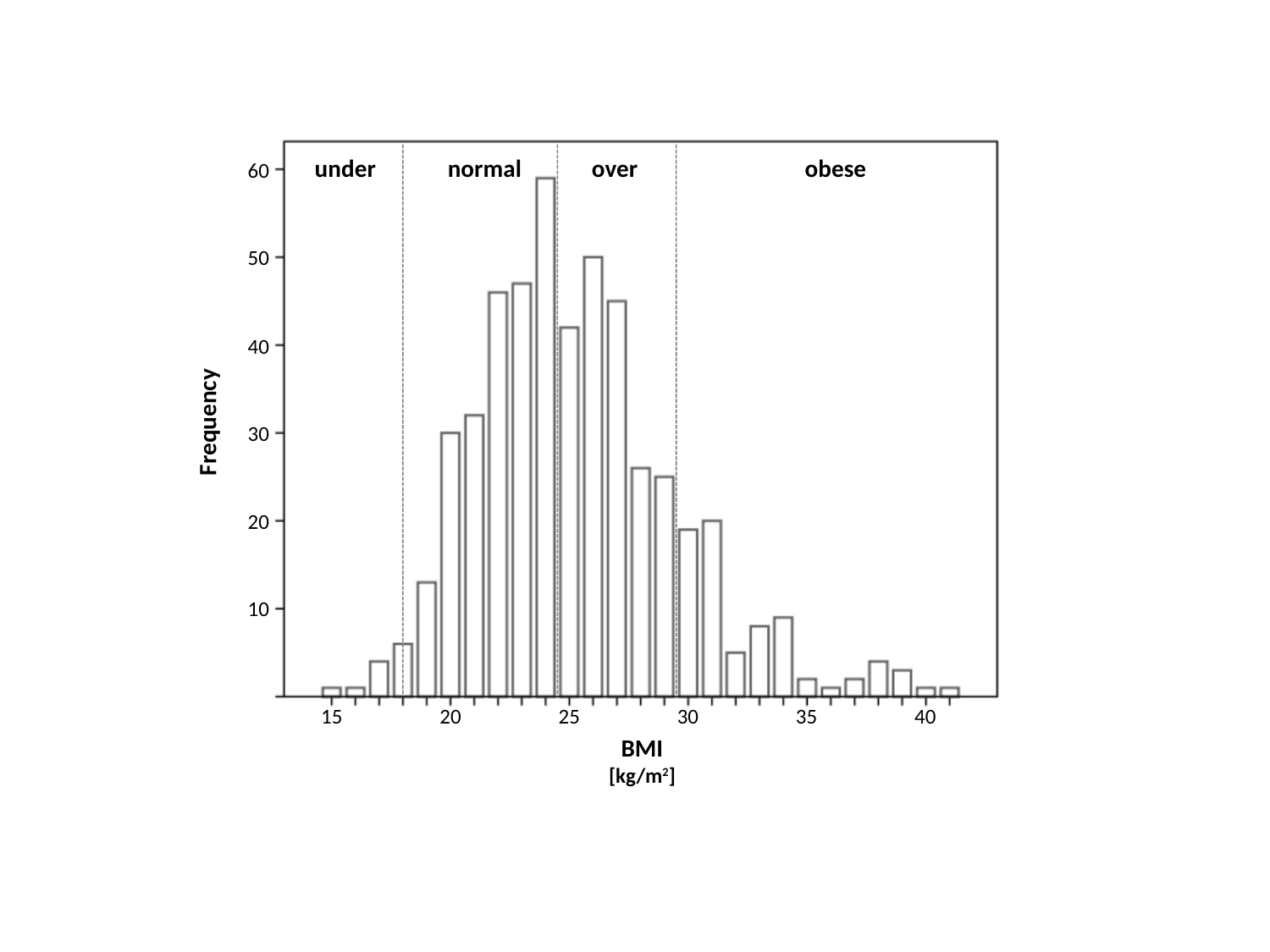

under
normal
over
obese
60
50
40
Frequency
30
20
10
15
20
25
30
35
40
BMI
[kg/m2]

Supplement: Supplementary file 1 — BMI distribution: Of 502 patients 1.2% were underweight with a BMI of <18.5 kg/m2 (under), 40.8% were of normal weight with a BMI of ≥18.5 – 24.9 kg/m2 (normal), 40.4% were overweight with a BMI of ≥25 – 29.9 kg/m2 (over) and 17.5% were obese with a BMI ≥30 kg/m2 (obese). (PPT 72 kb) [file 277_2017_3003_MOESM1_ESM.ppt]
